# Supplementary material for: Contacting domains segregate a lipid transporter from a solute transporter in the malarial host–parasite interface
Source: Nat Commun. 2020 Jul 30;11:3825. doi: 10.1038/s41467-020-17506-9 (PMC7393353; doi:10.1038/s41467-020-17506-9)
Supplement: Supplementary file 1 — Supplementary Information [file 41467_2020_17506_MOESM1_ESM.pdf]

Supplementary Information

Contacting domains segregate a lipid transporter from a solute transporter in the malarial host-parasite interface

Garten et al.

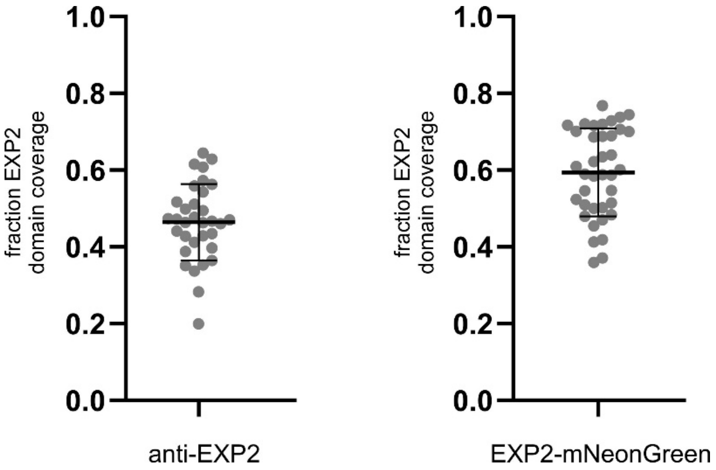

Supplementary Figure 1. Fraction of PVM covered by EXP2 determined by an indirect immuno-fluorescence assay and live cell fluorescence.

Fraction of EXP2 domain coverage (bars: mean+/-SD) in the projections determined by thresholding. The plot compares the domain coverage as measured by either NF54 antibody labeled against EXP2 (N=33 cells) and EXP2-mNeonGreen--PV-mRuby3 parasite line (N=38 cells). For both techniques about half of the parasite is covered with EXP2.

Source data are provided as a Source Data file.

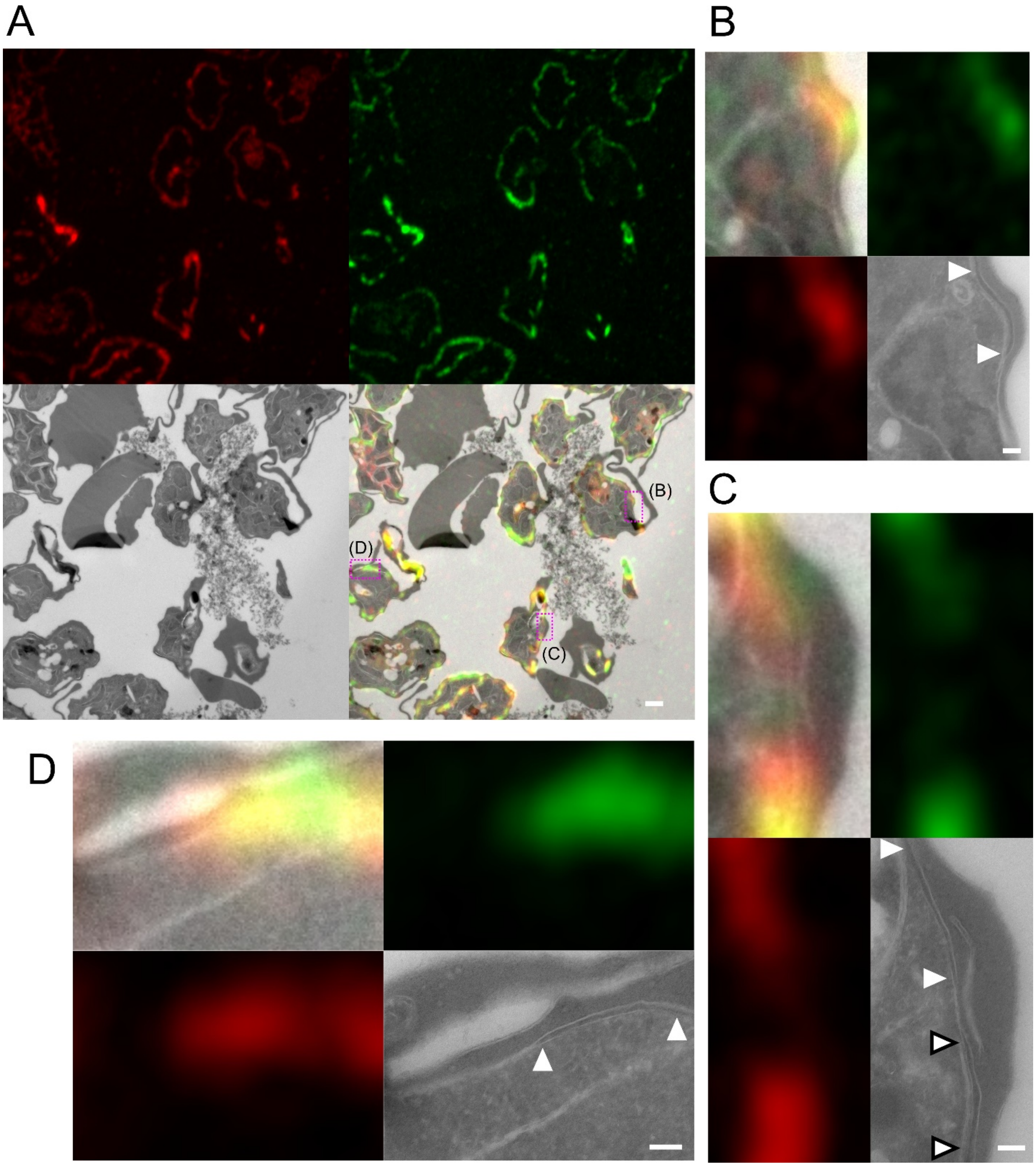

Supplementary Figure 2. Correlative light electron microscopy.
(A) Zoomed out view of the correlation shown in figure 3. Scale bar: 1 µm.
(B-D) Zoom into three regions as indicated by the magenta boxes in (A). Triangles outline regions of wider PV lumen, corresponding to fluorescent signal from PV-Ruby3. The gray image on the bottom right is from an image with higher magnification to allow examination of the PV structure. Panel (C) is featured in figure 3. Scale bar: 100 nm.
Red: PV-mRuby3, Green: EXP2-mNeonGreen, Gray: electron micrograph.

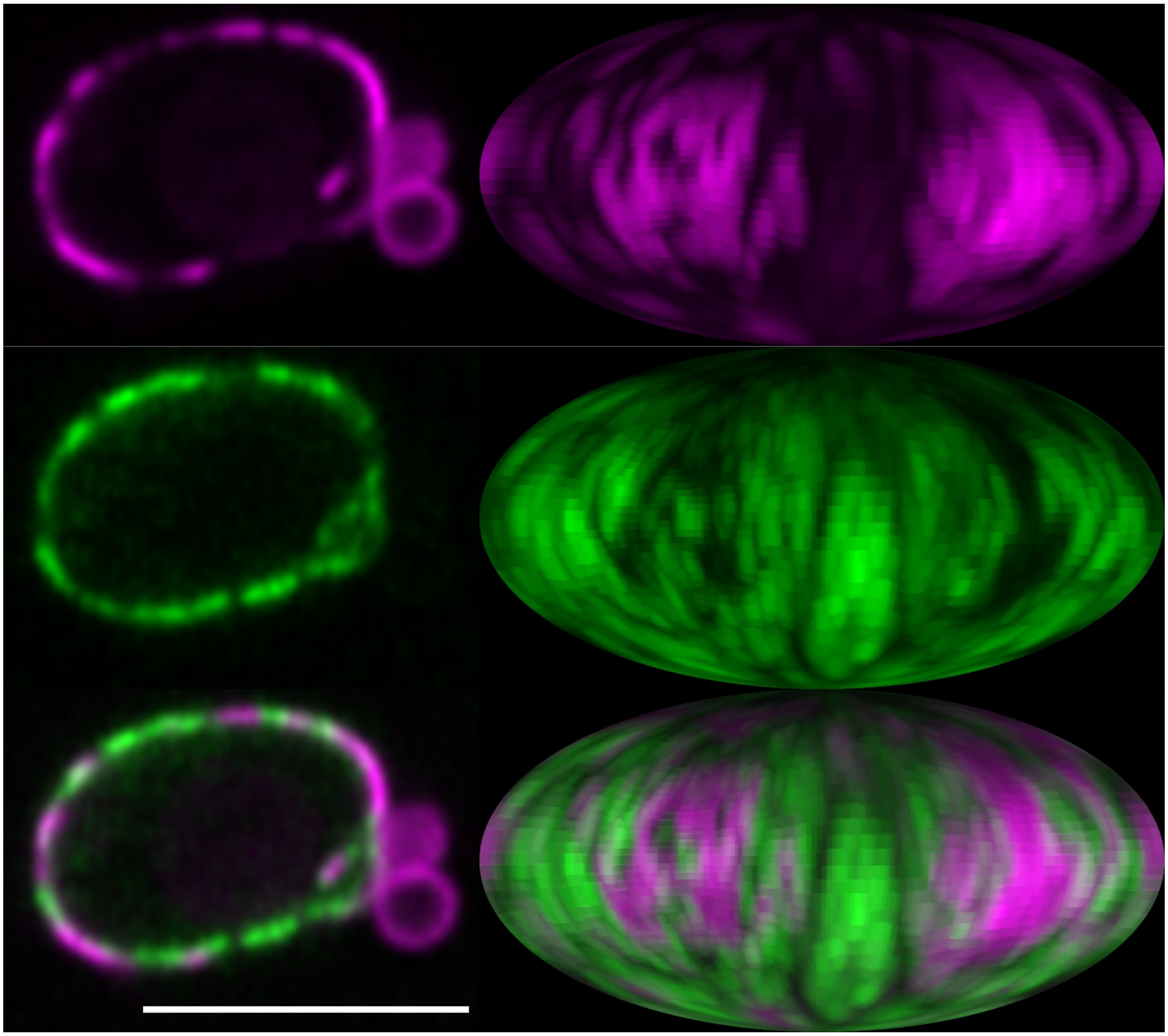

Supplementary Figure 3. Correlation of PV-mRuby3 and PfNCR1-GFP.
Central confocal slice and Mollweide projections of a parasite expressing PV-mRuby3 (magenta) and PfNCR1-GFP (green). The Pearson correlation coefficient for this cell is -0.11. Scale bar: 5 µm.

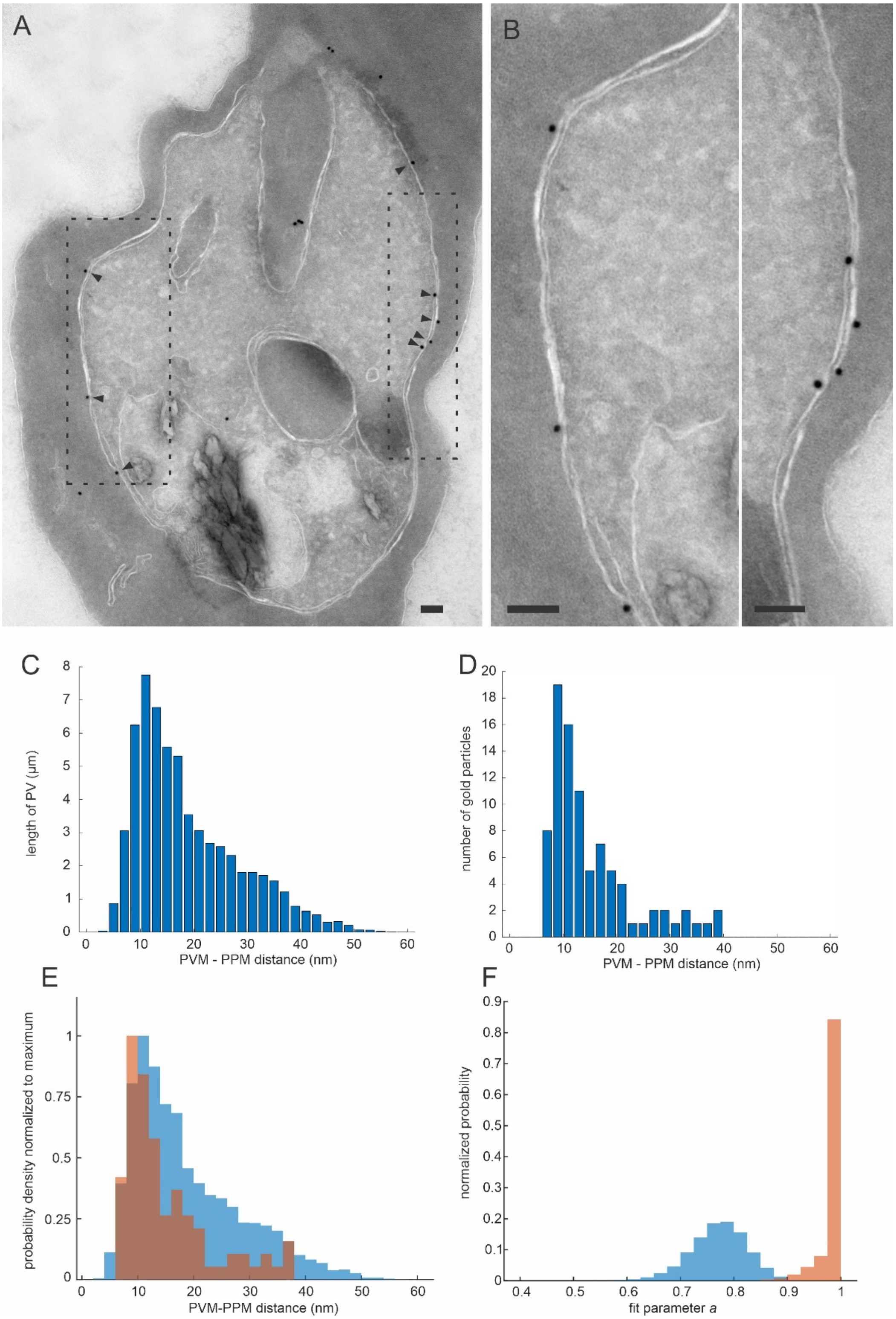

Supplementary Figure 4. Localization of PfNCR1-GFP in immunogold labeled Tokuyasu cryosections.

(A) Image showing a section of a trophozoite stage parasite. The 18 nm gold (black dots) labels PfNCR1-GFP. For experimental details see Istvan et al.[1]. Membranes are visible as white lines. Black arrow heads show gold label at the host-parasite interface.

(B) Zoom into two regions at the host-parasite interface. (A & B) scale bar: 100 nm.
(C) PVM-PPM distance of the Istvan et al. dataset containing 24 cells. Bootstrapping N = 1118 54 nm long PV segments, corresponding to the length in which a gold particle can bind to PfNCR1-GFP (see methods), yield as mixture model fit values (means [95% CI]): mu1 = 13.4 nm [12.2 nm, 14.5 nm], sigma1 = 1.51 nm [1.43 nm, 1.59 nm] and mu2 = 30.5 nm [27.4 nm, 33.3 nm], sigma 2 = 1.26 nm [1.19 nm, 1.34 nm], a = 0.77 [0.66, 0.86].
(D) Histogram of PVM-PPM distances found closest to a gold particle of the complete dataset. All particles in a distance of at most 18 nm (gold surface to membrane corresponding to the length of the primary and secondary antibody) from the PPM with identifiable PVM and PPM at the parasite periphery were measured. In the dataset of 24 cells, 88 gold particles fitting the criteria were identified. The relative contribution parameter *a* is 0.99 [0.93, 1.00] (means [95% CI], N=88 particles) using the mu and sigma values of all available PVM-PPM distances of the sample determined in (C). Thus, 99 % of the distribution can be described by the mu1 term of the mixture model.
(E) Histograms C (all membrane distances, blue) and D (PfNCR1-GFP gold label, orange) normalized to their maximum and plotted together. The Kolmogorov–Smirnov test statistic Young 1977 (see Supplementary reference [2]) is consistent with the hypothesis that the two distributions are significantly different.
(F) Distribution of the relative contribution of the first term to the mixture model (parameter *a* of the fit). Orange: a_{gold} of PfNCR1-GFP gold label, blue: a_{all} of all 54 nm PVM-PPM segments. Parameter *a* describes the abundance of close membrane distance, contrarywise (*a*-1) the abundance of larger membrane distance. Relative enrichment was calculated as (a_{gold} (1- a_{all})) / (a_{all} (1- a_{gold})): the gold label is 40.0x [14.6x, 61.4x] (mean [95% CI], error is propagated from the CI of the distributions) more likely to be found in the closely apposed membrane regions compared to the regions that show more PV lumen.
Source data are provided as a Source Data file.

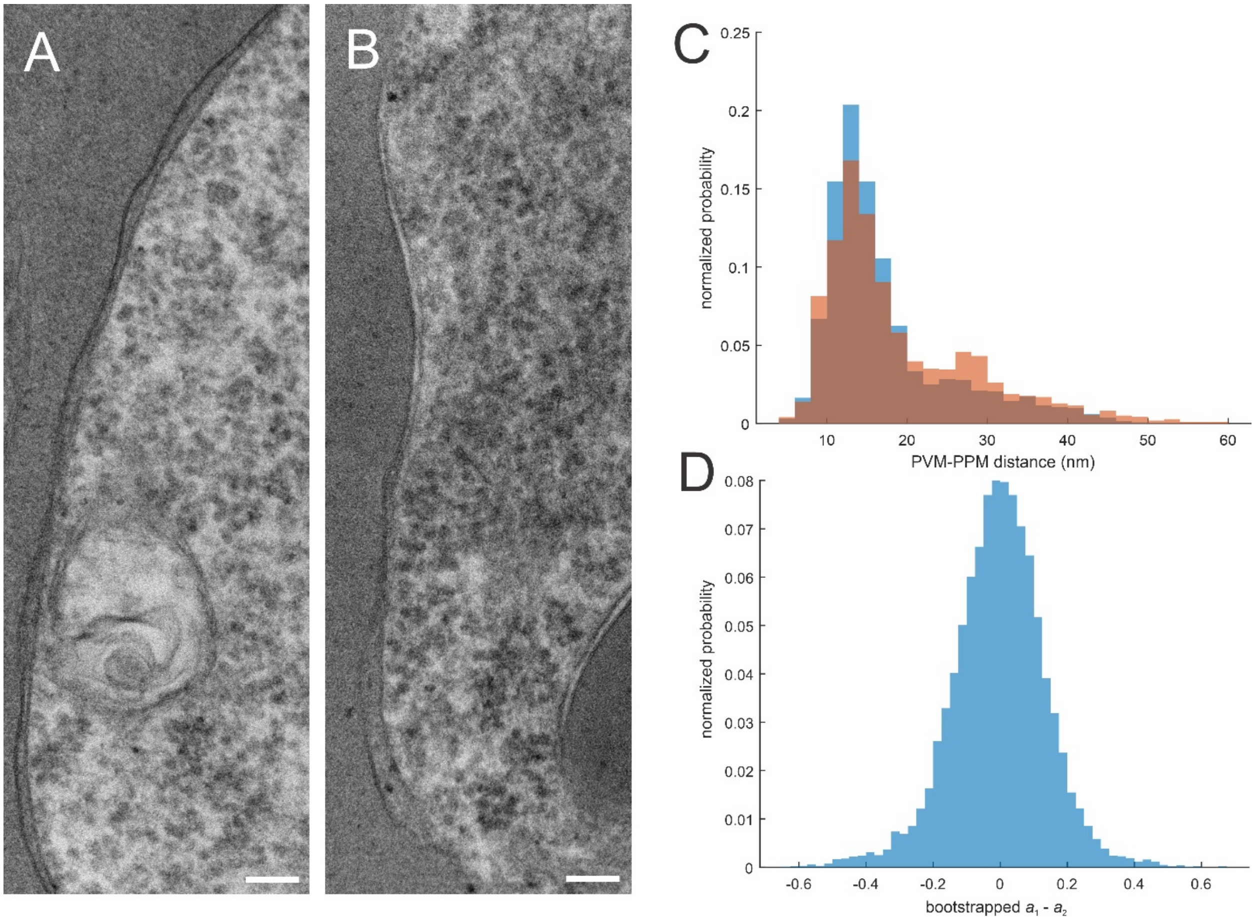

Supplementary Figure 5. PVM-PPM distance after PfNCR1-KD. Sample prepared for Istvan et al. 2019 [1].

(A) PfNCR1 knock-down parasite (fixation 68 hrs post anhydrotetracyline (aTc) wash-out).
(B) Control parasite, aTc was maintained. (A & B) scale bar: 100 nm.
(C) Histogram of membrane distances of the PfNCR1-knock-down (orange, N = 34 images) and its control (blue, N = 46 images). The distributions were bootstrapped using the two-component mixture model (see methods) yielding the following values (mean [95% CI]): Control: mean 1 = 13.3 nm [12.7 nm, 14.1 nm], mean 2 = 30.0 nm [25.4 nm, 34.0 nm], a = 0.80 [0.67, 0.91], PfNCR1 Knock-down: mean 1 = 13.1 nm [12.0 nm, 14.0 nm], mean 2 = 28.4 nm [24.3 nm, 31.1 nm], a = 0.66 [0.46, 0.83].
(D) Bootstrap calculation of the significance of the difference of relative contribution parameter a of the two components using a mixed (control and KD) sample. The 95% confidence interval of the difference of the simulated parameters is [-0.31 0.27]. The difference in the histogram in (C) is 0.14, thus, the PVM-PPM distance of the PfNCR1 knock-down and control are not significantly different.
Source data are provided as a Source Data file.

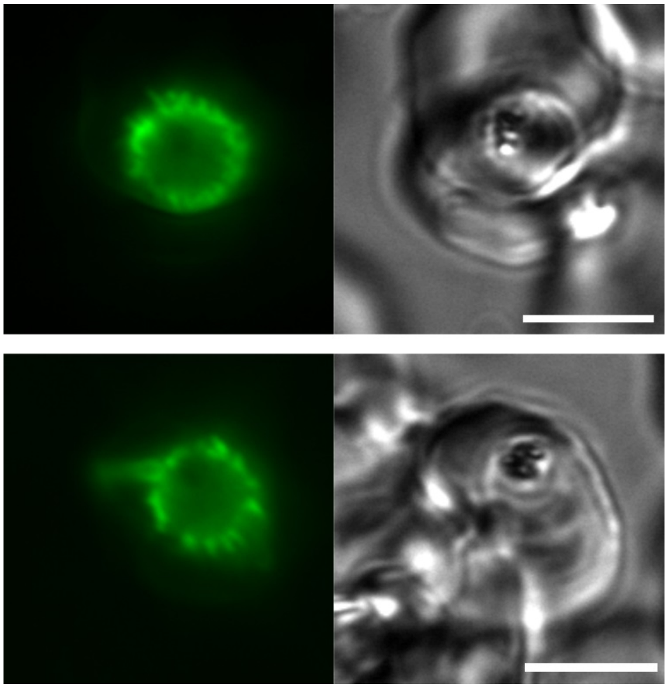

Supplementary Figure 6. EXP2 is targeted to distensions when protein export is impaired. Compared to the broken but smooth outline of EXP2-mNeonGreen in unmodified parasites (see figure 2) the outline after impairing protein export by inactivating HSP101 using a (DHFR)-based destabilization domain (DDD) leads to the appearance of a distended signal. This suggests that EXP2 is present in the distended membrane reported in Garten et al. [3].
Green: EXP2-mNeonGreen, gray: brightfield, scale bar: 5 µm.
Trimethoprim was withdrawn 24 hours before imaging. Image was acquired on an Axio Imager M1 equipped with a Plan-Apochomat 100x/1.4NA objective and ORCA-ER CCD camera, using the GFP filterset for the mNeonGreen imaging.

Primer name	Sequence
BSDinact	GCCAGCGCAGCTCTCTCTAGCGACGGGCGCATCTTCACTG GTGTCAATG
E2mRubyF	GATGAAAATAAAGAACCTAGGGGAAGTGGAGGAGTG
E2mRubyR	TAACTCGACGCGGCCGTCACTTGTACAGCTCGTCCATGCC
P150mRubyF	ACTTATCTAAGACCCCTAGGGGAAGTGGAGGAGTGTCTAA GGGCGAAGAGCTGATCAAGG
P150mRubyR	TAACTCGACGCGGCCGTCACTTGTACAGCTCGTCCATGCC
P150FLF	CACTATAGAACTCGAGGGTATAGAAAAAATATATAATATT TATATGCTTTTCTGCC
P150FLR	CTCCACTTCCCCTAGGGTTATCATCTTCTTCTTCGTCTAAT TCTTCTTCATC

Supplementary Table 1. Table of primers used.

Supplementary references

1. Istvan, E. S. *et al.* Plasmodium Niemann-Pick type C1-related protein is a druggable target required for parasite membrane homeostasis. *eLife* **8**, e40529 (2019).
2. Young, I. T. Proof without prejudice: use of the Kolmogorov-Smirnov test for the analysis of histograms from flow systems and other sources. *J. Histochem. Cytochem.* **25**, 935–941 (1977).
3. Garten, M. *et al.* EXP2 is a nutrient-permeable channel in the vacuolar membrane of Plasmodium and is essential for protein export via PTEX. *Nat. Microbiol.* **3**, 1090 (2018).
