# Supplementary figures and images for: Contacting domains segregate a lipid transporter from a solute transporter in the malarial host–parasite interface
Source: Nat Commun. 2020 Jul 30;11:3825. doi: 10.1038/s41467-020-17506-9 (PMC7393353; doi:10.1038/s41467-020-17506-9)

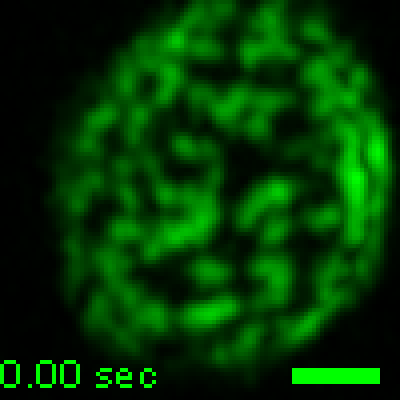

Supplement: Supplementary file 3 — Supplementary Movie 1 [file 41467_2020_17506_MOESM3_ESM.gif]

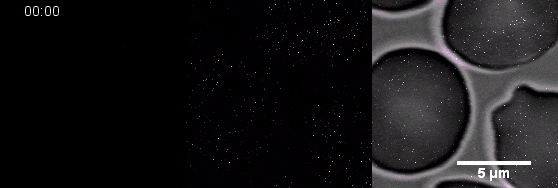

Supplement: Supplementary file 4 — Supplementary Movie 2 [file 41467_2020_17506_MOESM4_ESM.gif]
