# Supplementary material for: Contacting domains segregate a lipid transporter from a solute transporter in the malarial host–parasite interface
Source: Nat Commun. 2020 Jul 30;11:3825. doi: 10.1038/s41467-020-17506-9 (PMC7393353; doi:10.1038/s41467-020-17506-9)
Supplement: Supplementary file 5 — Description of Additional Supplementary Information [file 41467_2020_17506_MOESM5_ESM.pdf]

**Description of Additional Supplementary Files**

**File Name**: Supplementary Movie 1
**Description:** Dynamics of relatively small EXP2 domains. Time course of Z-projection of a stack of the PVM face closest to the cover slide. Green: EXPmNeonGreen. Scale bar: 1 µm.

**File Name:** Supplementary Movie 2
**Description:** Plasmodium intra-erythrocytic lifecycle. This movie demonstrates the domain variability in the P. falciparum life cycle. Green: EXP2-mNeonGreen, magenta: PTEX150-mRuby3, gray: bright field image. Time stamp shows hours : minutes.
