# Supplementary material for: Contacting domains segregate a lipid transporter from a solute transporter in the malarial host–parasite interface
Source: Nat Commun. 2020 Jul 30;11:3825. doi: 10.1038/s41467-020-17506-9 (PMC7393353; doi:10.1038/s41467-020-17506-9)
Supplement: Supplementary file 6 — Reporting Summary [file 41467_2020_17506_MOESM6_ESM.pdf]

nature research

Corresponding author(s): Joshua Zimmerberg
Daniel E. Goldberg

Last updated by author(s): Jun 17, 2020

Reporting Summary

Nature Research wishes to improve the reproducibility of the work that we publish. This form provides structure for consistency and transparency in reporting. For further information on Nature Research policies, see Authors & Referees and the Editorial Policy Checklist.

Statistics

For all statistical analyses, confirm that the following items are present in the figure legend, table legend, main text, or Methods section.

n/a	Confirmed	
☐	☒	The exact sample size (n) for each experimental group/condition, given as a discrete number and unit of measurement
☐	☒	A statement on whether measurements were taken from distinct samples or whether the same sample was measured repeatedly
☐	☒	The statistical test(s) used AND whether they are one- or two-sided<br>*Only common tests should be described solely by name; describe more complex techniques in the Methods section.*
☒	☐	A description of all covariates tested
☒	☐	A description of any assumptions or corrections, such as tests of normality and adjustment for multiple comparisons
☐	☒	A full description of the statistical parameters including central tendency (e.g. means) or other basic estimates (e.g. regression coefficient) AND variation (e.g. standard deviation) or associated estimates of uncertainty (e.g. confidence intervals)
☐	☒	For null hypothesis testing, the test statistic (e.g. F, t, r) with confidence intervals, effect sizes, degrees of freedom and P value noted<br>*Give P values as exact values whenever suitable.*
☒	☐	For Bayesian analysis, information on the choice of priors and Markov chain Monte Carlo settings
☒	☐	For hierarchical and complex designs, identification of the appropriate level for tests and full reporting of outcomes
☐	☒	Estimates of effect sizes (e.g. Cohen's d, Pearson's r), indicating how they were calculated

Our web collection on statistics for biologists contains articles on many of the points above.

Software and code

Policy information about availability of computer code

Data collection	Zen Black, AMT image capture engine
Data analysis	ImageJ 1.52p, Matlab 2018b, Matlab 2019b, eC-CLEM, Prism

For manuscripts utilizing custom algorithms or software that are central to the research but not yet described in published literature, software must be made available to editors/reviewers. We strongly encourage code deposition in a community repository (e.g. GitHub). See the Nature Research guidelines for submitting code & software for further information.

Data

Policy information about availability of data

All manuscripts must include a data availability statement. This statement should provide the following information, where applicable:

- Accession codes, unique identifiers, or web links for publicly available datasets
- A list of figures that have associated raw data
- A description of any restrictions on data availability

The data that support the findings of this study are available from the corresponding authors on request.

Field-specific reporting

Please select the one below that is the best fit for your research. If you are not sure, read the appropriate sections before making your selection.

☒ Life sciences ☐ Behavioural & social sciences ☐ Ecological, evolutionary & environmental sciences

For a reference copy of the document with all sections, see nature.com/documents/nr-reporting-summary-flat.pdf

Life sciences study design

All studies must disclose on these points even when the disclosure is negative.

Sample size	no sample size calculation was performed
Data exclusions	only trophozoite stage parasites (undivided cell, containing Haemozoin) were considered, only parasites with fluorescent signal were considered for the analysis of fluorescence
Replication	Fluorescence data shown was collected as single larger data sets of observations made previously in at least 3 different smaller sets in blood from different donors. Correlative light electron microscopy was performed twice on the EXP2-mNeonGreen--PV-mRuby3 line. Freeze fracture was performed multiple times. Findings are consistently replicated with different techniques as described in the manuscript (live cell, chemically fixed, frozen, different electron microscopy preparations).
Randomization	Cells for the correlation analysis were chosen from a bright field image
Blinding	Investigators were not blinded as they prepared, performed and analyzed experiments with the exception of acquisition of images showing immuno-gold label.

Reporting for specific materials, systems and methods

We require information from authors about some types of materials, experimental systems and methods used in many studies. Here, indicate whether each material, system or method listed is relevant to your study. If you are not sure if a list item applies to your research, read the appropriate section before selecting a response.

Materials & experimental systems

n/a	Involved in the study
☐	☒ Antibodies
☐	☒ Eukaryotic cell lines
☒	☐ Palaeontology
☒	☐ Animals and other organisms
☐	☒ Human research participants
☒	☐ Clinical data

Methods

n/a	Involved in the study
☒	☐ ChIP-seq
☒	☐ Flow cytometry
☒	☐ MRI-based neuroimaging

Antibodies

Antibodies used	mouse anti-EXP2 mAb clone 7.7 (Hall et al., (1983) Mol. Biochem Parasitol. 7: 247-265, The European Malaria Reagent Repository) (IFA 1:500)<br>Donkey-anti-mouse conjugated with Alexa Fluor 488, Invitrogen A21202, lot 1113537, 1:150)<br>Antibodies and sample preparation of the immuno-gold label were described in Istvan et al. eLife 8:e40529 (2019)
Validation	anti-EXP2 is validated for example in Garten, M. et al. Nat. Microbiol. 3:1090 (2018) when used with the conditional EXP2 knock-down

Eukaryotic cell lines

Policy information about cell lines

Cell line source(s)	The Plasmodium falciparum NF54attB line used in this study was obtained from the Fidock lab where it was generated. NF54 was obtained from BEI Resources
Authentication	PCR amplified regions from the NF54attB genome were found to match the genome sequence for 3D7, a sub clone of NF54. The prescence of the cg6 localized attB sequence was verified by successful Bxb1 integration at that site.
Mycoplasma contamination	Mycoplasma contamination was tested occasionally
Commonly misidentified lines (See ICLAC register)	No commonly misidentified lines were used in the study.

Human research participants

Policy information about studies involving human research participants

Population characteristics	parasites were cultured in human red blood cells obtained from de-identified donors

Population characteristics	participating in the NIH IRB-approved Research Donor Program in Bethesda, MD.
Recruitment	Donors are selected by the NIH blood bank. The authors had no influence on the selction.
Ethics oversight	The NIH Institutional Review Board approved the program

Note that full information on the approval of the study protocol must also be provided in the manuscript.
